# Supplementary material for: DMRforPairs: identifying Differentially Methylated Regions between unique samples using array based methylation profiles
Source: BMC Bioinformatics. 2014 May 15;15:141. doi: 10.1186/1471-2105-15-141 (PMC4046028; doi:10.1186/1471-2105-15-141)
Supplement: Additional file 2 — DMRforPairs output for the comparison of A431-MCF7 and NA17018-NA17105. Please start from the HTML files in each folder. Available via the BMC Bioinformatics website. [file 1471-2105-15-141-S2.zip › 1394847754114233_MOESM2_ESM/A431_MCF7/figures/28849.pdf]

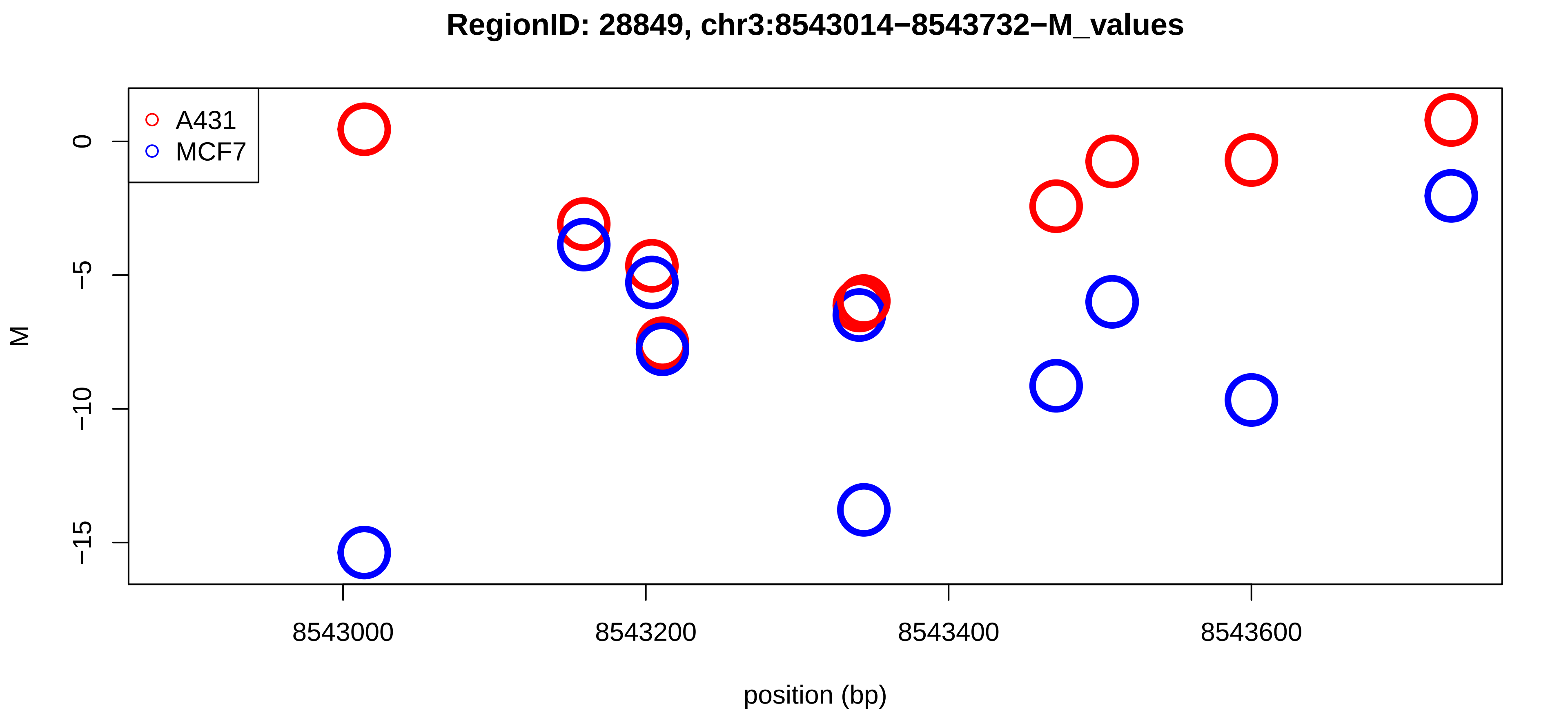

RegionID: 28849, chr3:8543014−8543732−M_values
A431
MCF7
M
0
−5
−10
−15
8543000
8543200
8543400
8543600
position (bp)

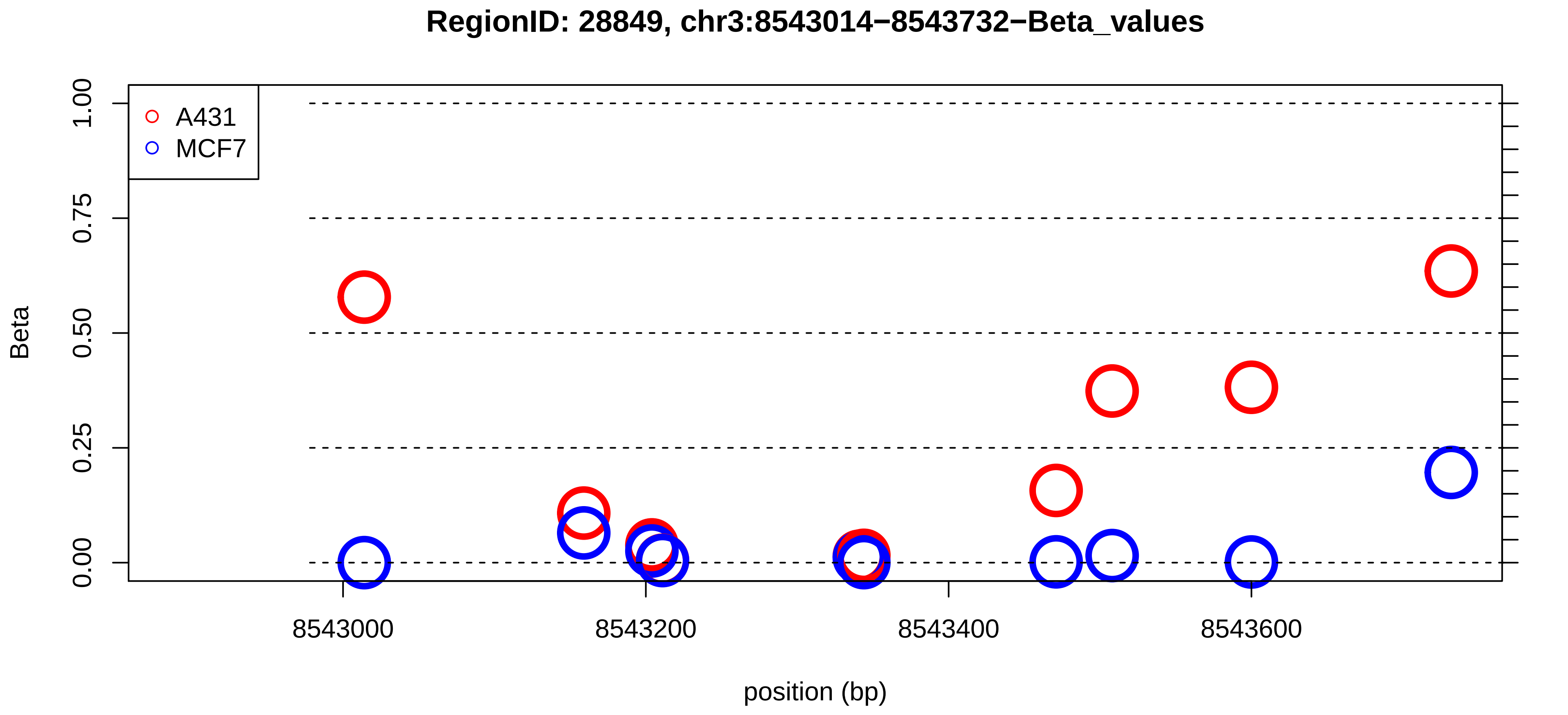

RegionID: 28849, chr3:8543014−8543732−Beta_values
A431
MCF7
Beta
0.00
0.25
0.50
0.75
1.00
8543000
8543200
8543400
8543600
position (bp)

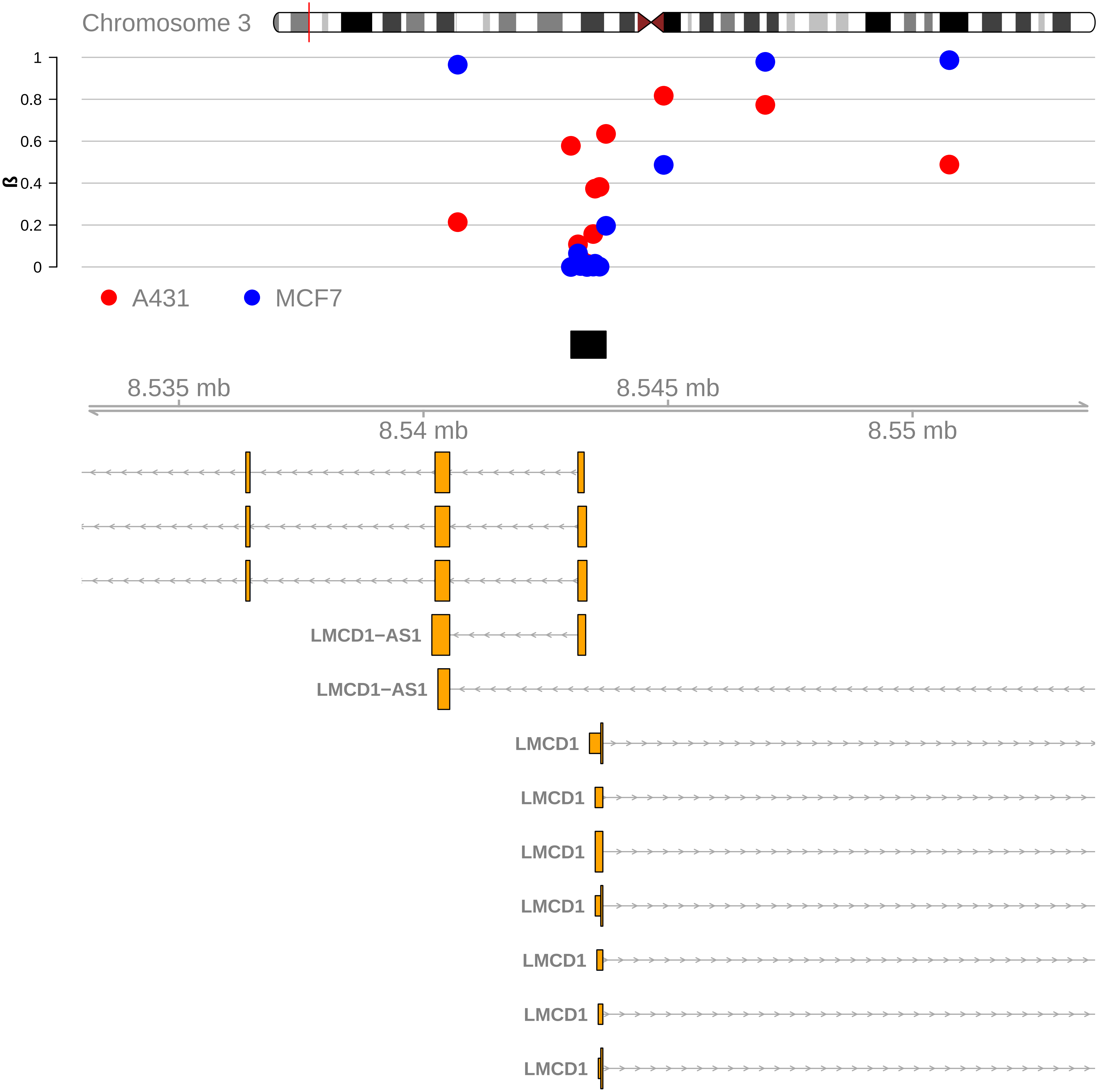
Chromosome 3
ß
1
0.8
0.6
0.4
0.2
0
A431
MCF7
8.535 mb
8.545 mb
8.54 mb
8.55 mb
LMCD1−AS1
LMCD1−AS1
LMCD1
LMCD1
LMCD1
LMCD1
LMCD1
LMCD1
LMCD1
